# Feasibility of a multimodal exercise, nutrition, and palliative care intervention in advanced lung cancer

**DOI:** 10.1186/s12885-021-07872-y

**Published:** 2021-02-13

**Authors:** Manuel Ester, S. Nicole Culos-Reed, Amane Abdul-Razzak, Julia T. Daun, Delaney Duchek, George Francis, Gwyn Bebb, Jennifer Black, Audra Arlain, Chelsia Gillis, Lyle Galloway, Lauren C. Capozzi

**Affiliations:** 1 Faculty of Kinesiology, University of Calgary, Calgary, Alberta Canada; 2 Department of Oncology, Cumming School of Medicine, University of Calgary, Calgary, Alberta Canada; 3 Department of Psychosocial Resources, Tom Baker Cancer Centre, Calgary, Alberta Canada; 4 Division of Palliative Medicine, Department of Oncology, University of Calgary, Calgary, Alberta Canada; 5 Division of Physical Medicine and Rehabilitation, Department of Clinical Neurosciences, Cumming School of Medicine, University of Calgary, Calgary, Alberta Canada; 6 Cancer Control Alberta, Alberta Health Services, Calgary, Alberta Canada; 7 Nutrition Services, Foothills Medical Centre, Cancer Care & Alberta Healthy Living Program, Calgary, Alberta Canada; 8 Community Health Sciences, University of Calgary, Calgary, Alberta Canada; 9 Cumming School of Medicine, University of Calgary, Calgary, Alberta Canada; 10 Department of Family Medicine, Cumming School of Medicine, University of Calgary, Calgary, Alberta Canada

**Keywords:** Exercise oncology, Supportive cancer care, Quality of life, Advanced lung cancer, Nutrition, Symptom management

## Abstract

**Background:**

Advanced lung cancer patients face significant physical and psychological burden leading to reduced physical function and quality of life. Separately, physical activity, nutrition, and palliative symptom management interventions have been shown to improve functioning in this population, however no study has combined all three in a multimodal intervention. Therefore, we assessed the feasibility of a multimodal physical activity, nutrition, and palliative symptom management intervention in advanced lung cancer.

**Methods:**

Participants received an individually tailored 12-week intervention featuring in-person group-based exercise classes, at-home physical activity prescription, behaviour change education, and nutrition and palliative care consultations. Patients reported symptom burden, energy, and fatigue before and after each class. At baseline and post-intervention, symptom burden, quality of life, fatigue, physical activity, dietary intake, and physical function were assessed. Post-intervention interviews examined participant perspectives.

**Results:**

The multimodal program was feasible, with 44% (10/23) recruitment, 75% (75/100) class attendance, 89% (8/9) nutrition and palliative consult attendance, and 85% (17/20) assessment completion. Of ten participants, 70% (7/10) completed the post-intervention follow-up. Participants perceived the intervention as feasible and valuable. Physical activity, symptom burden, and quality of life were maintained, while tiredness decreased significantly. Exercise classes prompted acute clinically meaningful reductions in fatigue, tiredness, depression, pain, and increases in energy and well-being.

**Conclusion:**

A multimodal physical activity, nutrition, and palliative symptom management intervention is feasible and shows potential benefits on quality of life that warrant further investigation in a larger cohort trial.

**Trial registration:**

NCT04575831, Registered 05 October 2020 – Retrospectively registered.

**Supplementary Information:**

The online version contains supplementary material available at 10.1186/s12885-021-07872-y.

## Background

In 2020, nearly 30,000 Canadians will be diagnosed with lung cancer, making it the most commonly diagnosed cancer in Canada [1]. As a result of improved treatment, including modern targeted therapy and immuno-therapy, lung cancer survival rates are steadily increasing, with roughly one in five patients surviving at least 5 years [1]. The number living with advanced or incurable disease is also on the rise. Unfortunately, this prolonged survival leaves a growing number of patients with ongoing psychological and physical treatment and disease related burden. Specifically, these patients face increased rates of anxiety and depression, impaired physical health, and increased sedentary behavior; all contributing to reduced quality of life (QOL) [2, 3].

Research suggests that physical function and physical independence are central to improving QOL among the growing number of advanced lung cancer (ALC) survivors [4]. Three key interventions have been found to positively impact patient outcomes, including physical activity (PA), nutritional support, and palliative symptom management [5–8]. PA positively impacts physical and mental well-being in advanced cancer, while improved nutritional status is correlated with improved QOL among these patients [7, 9]. Palliative medicine can address debilitating symptoms uniquely challenging ALC patients, including pain and shortness of breath [10].

Evidence supports the use of PA, nutrition, and palliative symptom management as separate interventions for enhancing QOL in ALC. However, few studies have examined their potential synergistic effects. A randomized controlled trial (RCT) of an exercise and nutrition intervention for advanced cancer, including non-small cell lung cancer (NSCLC) patients, showed that a bimodal intervention is feasible in this population [11]. Over the 3-month intervention, retention rates were 72% and supervised exercise session attendance was 75% [11]. Intervention effectiveness was mixed however, with improvements in symptom burden and protein intake but no change in physical fitness or overall QOL [11]. Current evidence on the feasibility of combined PA and nutrition interventions in advanced cancer is inconclusive, and no trials to date have integrated palliative symptom management into a multimodal intervention for this population [8].

Therefore, the primary aim of this study was to assess feasibility of a novel 12-week trimodal intervention, including PA, nutrition and palliative symptom management in advanced NSCLC, an underserved advanced cancer population. Feasibility was defined a priori as at least 30% recruitment, 60% attendance, 70% assessment completion, and no adverse events. These percentages were based on clinical consultations, ongoing exercise oncology studies, and previous work in advanced cancer [12, 13]. Secondary aims were to assess intervention impact on patient reported QOL, PA, symptom burden, and physical function. We hypothesized that the PA and Exercise, Nutrition, and Palliative care in ALC, or “‘ENPAL” intervention would be feasible and would demonstrate preliminary efficacy for enhancing QOL.

## Methods

### Participant recruitment and eligibility

All participants were recruited in-person at the lung cancer clinic at the Holy Cross Centre (Calgary, Alberta). Inclusion criteria were: 1) 18+ years old; 2) stage III-IV NSCLC; 3) Eastern Cooperative Oncology Group (ECOG) performance status 0–2; 4) Edmonton Symptom Assessment System (ESAS) score ≥ 3/10 on at least one item; 5) hemoglobin ≥80 g/L; 6) life expectancy > 6 months; and 7) cleared for exercise by an oncologist. Exclusion criteria were: 1) active infections; 2) enteral tube feeding or parenteral nutrition; 3) mechanical or functional bowel obstruction; 4) cognitive impairment; and 5) non-English speaking. Eligible participants were briefed by their oncologist before receiving additional study information from the study team upon providing permission. Those who were interested attended the pre-intervention assessment to complete informed consent and required measures.

### Study design and intervention

The current mixed-methods study used a prospective design, with participants receiving a multimodal intervention including PA, nutrition, and palliative symptom management. The PA intervention included an individualized plan which incorporated in-person group exercise class as well as a tailored at-home PA prescription. A PA prescription was developed based on the individual’s prior PA history, current physical function, current clinical condition, and with the goal of working towards the ACSM Cancer Exercise guidelines of 90 min of moderate intensity physical activity per week plus two sessions of resistance exercise and flexibility exercise most days of the week [14]. The starting at-home prescription recommended 2–4 days of light to moderate intensity aerobic exercise (i.e. walking), for approximately 10–30 min per session, with 1–2 days of resistance exercise using body weight, and an exercise ball or band (provided). The in-person exercise class, led by a clinical exercise physiologist, included individually tailored aerobic, resistance, and flexibility exercises. Resistance exercises targeting major muscle groups were performed for 2–3 sets of 8–10 repetitions. The program was progressed at 4, 6, 8 and 10 weeks (additional resistance/sets/repetitions or progression to a more challenging exercise) with the goal of progressing participants towards the ACSM Cancer Exercise Guidelines [14]. A sample of the exercise programs provided to ENPAL participants is included in Additional File 1.

Six educational workshops focused on health behaviour change and maintenance were integrated into exercise classes. Topics included: principles of exercise in cancer, goal-setting, behaviour change and relapse prevention, stress management and sleep, nutrition, and social support and long-term maintenance. Each topic targeted one of the following behavior change techniques according to the CALO-RE taxonomy: “information provision to the individual” (physical activity), “goal setting (behavior)”, “relapse prevention”, “stress management”, “information provision to the individual” (nutrition), and “plan social support” [15]. The nutrition and palliative care components included open-ended consultations with a cancer centre dietitian and palliative care physician to discuss patient needs. Participants were able to request follow-up as needed. Initial consults were scheduled within 2 weeks of exercise class start, with follow-up appointments arranged at the discretion of the patient and/or provider. Intervention duration was between 12 and 14 weeks, as participants were afforded additional time to complete up to 12 in-person exercise sessions. The Health Research Ethics Board of Alberta – Cancer Committee approved this study (HREBA.CC-18-0681).

### Assessments

Participants were assessed at pre-intervention and post-intervention (week 12–14). Assessments included questionnaires, physical function testing (symptom limited), and completion of a 24-h dietary recall. The pre-intervention assessment featured an informed consent discussion and demographic/medical history questionnaires. Optional post-intervention interviews were conducted during the second assessment. Clinical exercise physiologists conducted physical function testing. Semi-structured interviews were conducted by the lead author (ME). Assessment duration ranged between one to 2 h. Patient-reported fatigue, energy, and symptom burden was also collected before and after each class.

### Outcomes

#### Demographics

Demographics/medical history were obtained via baseline questionnaires. This included age, sex, marital status, education level, annual family income, employment status, cancer diagnosis and tumour marker status, current and previous treatment, current side effects, concurrent treatments and medications, past surgeries and musculoskeletal injuries, co-morbidities, and allergies. The medical information was used to inform safe exercise prescription.

#### Feasibility

Feasibility thresholds were determined a priori based on clinical consultations with senior members of the lung cancer clinic, ongoing exercise oncology studies, and previous work in advanced cancer [12, 13]. Recruitment data (number of eligible patients, reasons for ineligibility, reasons for non-participation when eligible) was collected during weekly recruitment visits. Participant attendance to assessments, weekly exercise classes, and the nutrition and palliative consults was recorded. Adherence to home-based exercise prescriptions and any other PA was monitored via paper-based weekly self-report journals. Exercise class attendance was based on the number of classes attended by all participants divided by the total number of classes offered (the sum of attended and missed classes).

#### Patient reported outcomes (PROs)

##### Physical activity

Due to unexpected shipping delays, Fitbit devices arrived after the start of the trial and could not be used for objective PA tracking from baseline onwards. PA was therefore measured using self-report via the modified Godin Leisure Time Exercise Questionnaire (GLTEQ), which has been validated for use in adult cancer populations [16]. Participants self-reported weekly PA frequency and duration across four categories: strenuous, moderate, mild, and resistance exercise. Weekly moderate-strenuous PA (MS PA) and total PA was calculated by multiplying frequency by duration in each category. The leisure score index (LSI) was calculated according to GLTEQ guidelines based on reported mild, moderate, and strenuous PA [16]. LSI is an estimate of weekly metabolic equivalent of task (MET) PA, classified as insufficiently active/sedentary (< 14), moderately active (14–23), or active (24+).

##### Symptom burden, fatigue, and quality of life – pre/post intervention

QOL was assessed using the validated Functional Assessment of Cancer Therapy – Lung (FACT-L), ESAS, and Functional Assessment of Chronic Illness Therapy – Fatigue (FACIT-F) questionnaires [17–19]. The FACT-L assesses physical (PWB), social/family (SWB), emotional (EWB), and functional (FWB) well-being as well as a lung cancer subscale (LCS). These were combined according to FACT-L scoring guidelines to yield an overall QOL score (FACT-L Total). The ESAS was used to measure symptom burden, and the FACIT-F to measure fatigue as two additional aspects of QOL. FACIT-F scores range from 0 to 52 and FACT-L total scores range from 0 to 136, with higher scores indicating better QOL.

##### Symptom burden, fatigue, and energy – pre/post class

Directly before and after each exercise class, participants reported ESAS symptom burden as well as fatigue and energy on single-item thermometers from 0 to 10. A single-item fatigue score was used instead of the FACIT-F to reduce participant burden. Acute effects of the class were assessed by comparing pre- and post-class means.

##### Nutrition

Dietary intake was assessed pre- and post-intervention via the Automated Self-Administered 24-h (ASA-24) dietary recall tool, completed with the study team for simplicity [20]. Participants were asked to recall all food or drink consumed within the past 24 h, including dietary supplements, which was then entered into the ASA24 system to yield automatically calculated nutrient intakes. The recall included a question assessing if the daily intake was typical for participants. Nutritional outcomes of interest were macronutrients and total calories per kilogram. Due to the pilot nature of intervention, a single day was collected to assess feasibility of performing the dietary recall.

#### Physical function

Physical function tests were conducted by a clinical exercise physiologist, who was not involved with intervention delivery. The same clinical exercise physiologist was responsible for pre- and post-intervention testing. Resting heart rate was measured using a 15-s pulse at the radial artery, multiplied by four to determine beats per minute. Resting blood pressure (mmHg) was measured in duplicate on the left arm using a sphygmomanometer and stethoscope using standardized procedures. Height and weight were collected on a Health Carter balance beam scale and used to calculate body mass index (BMI). While standing, participant waist circumference was measured with an anthropometric tape measure at the top of the iliac crest and hip circumference at the greatest gluteal girth [21]. Upper body strength was assessed using a handgrip dynamometer (Smedley Dynamometer, TTM, Tokyo, Japan), taking the average of two trials according to the Canadian Physical Activity Fitness and Lifestyle Approach (CPFLA) protocol [22]. Lower body strength was assessed using the 30-s sit-to-stand test, recording the number of times that participants could stand from a seated position in 30-s [23]. Shoulder range of motion was averaged from duplicate measurements using a goniometer. Trunk and leg flexibility was averaged from duplicate measurements using the sit-and-reach test (Wells-Dillon flexometer) in accordance with the CPAFLA protocol [16]. Single leg balance was measured using a standardized protocol by Fleishman [24]. A six-minute walk test was used to determine aerobic capacity, with walking distance around a 200-m track recorded to the nearest 0.5 m [22]. The six-minute walk test was limited to a single trial to reduce participant burden and the likelihood of eliciting negative symptoms such as shortness of breath.

#### Nutrition and palliative consult report forms

Information provided during nutrition and palliative consults was recorded using standardized case report forms for each respective component, with specific sections and checkboxes to streamline data summarization. The case report forms covered recommendations provided, issues discussed, and interventions suggested.

### Data analysis

#### Quantitative

Descriptive statistics were generated for all variables, with means and standard deviations for continuous variables and raw numbers, as well as percentages for categorical variables. Outcome values were inspected for normality to ensure that appropriate statistical tests were applied. Dependent sample t-tests were used to compare pre and post means for the intervention and individual exercise classes (to assess acute exercise class effects). *P*-values are reported for all t-tests, with a cut-off of *p* < 0.05 for statistical significance. Due to the preliminary nature of these analyses and the relatively small number of pre-planned comparisons, no correction for multiple comparisons was performed. All statistical tests were performed using IBM SPSS statistics 26.0 software (IBM).

#### Qualitative

Interpretive description, a commonly used methodology for qualitative health research, was used, to develop a deeper understanding of study feasibility and impact [25]. Data collection and analyses were guided by a constructivist philosophy, acknowledging that multiple social realities exist [26]. Interviews were transcribed verbatim by one author (DD) before proof-reading by the lead author (ME). Transcripts were then read by a third author (JD), who developed transcript notes for each interview and performed all coding independently, consistent with constructivist philosophy, using NVivo 12.0 software (QSR International). Four authors (JD, ME, LCC, NCR) iteratively grouped codes into themes based on common concepts, research questions, quantitative data, and theoretical knowledge in exercise oncology. The author who coded data (JD) then selected representative quotes for each theme. Readability was enhanced by removing stutters or repetitive words, replacing long pauses or tangent thoughts by “[...]”, and inserting words or replacing names by titles in square brackets.

## Results

### Feasibility and demographics

**Fig. 1:**
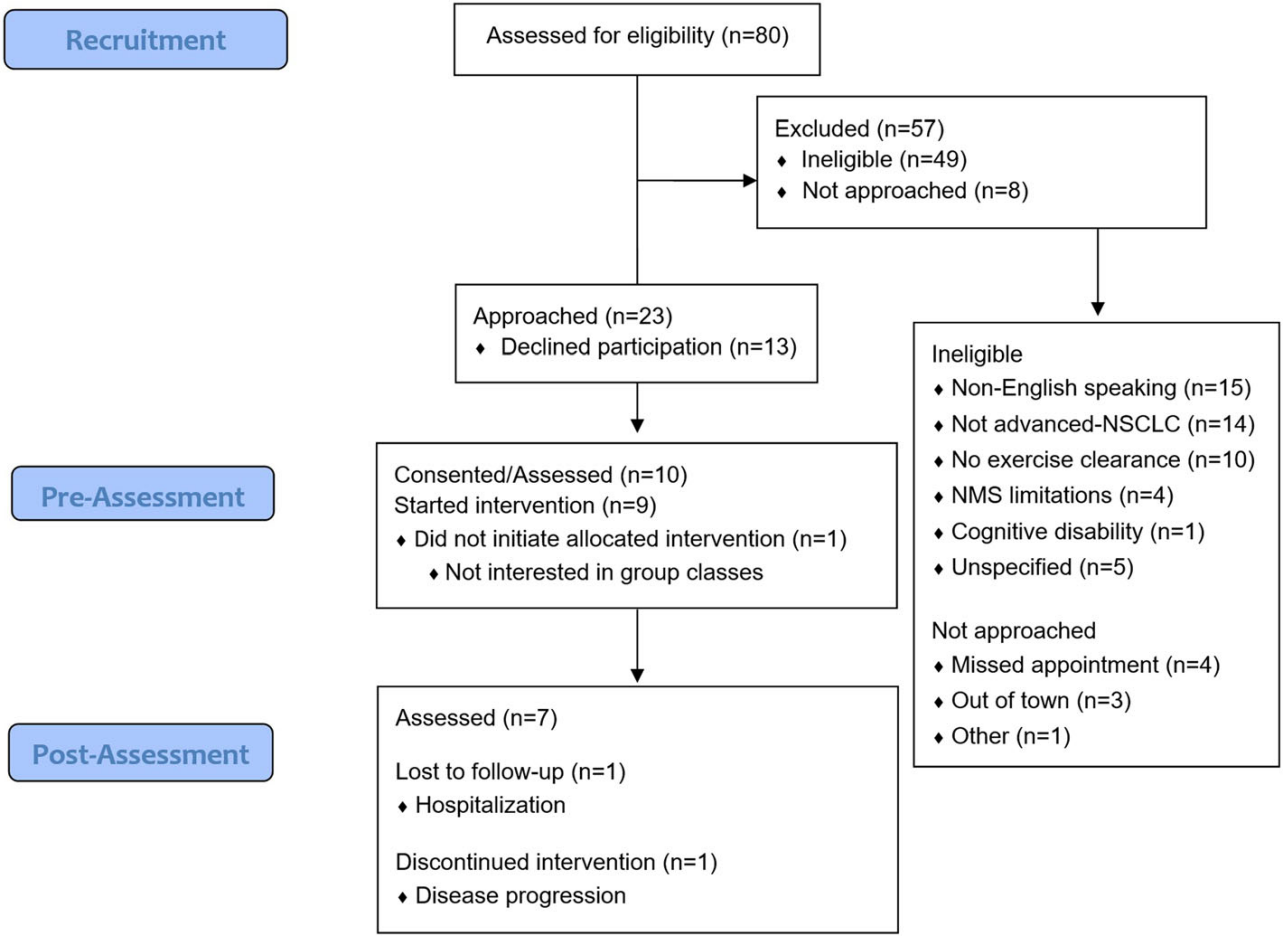
Modified CONSORT flow diagram for the single-arm ENPAL study. legend: Recruitment began in October 2019 and the post-assessments concluded in March 2020. NSCLC = non-small-cell lung cancer. NMS = neuromusculoskeletal

Over the nine-week recruitment period, 80 lung cancer patients were assessed for eligibility. Of the 31 who were eligible, 23 were approached, and 10 (43.5% of approached) provided informed consent (Fig. 1). One participant dropped out before starting the intervention due to lack of interest in group-based classes. As such, only the 9 remaining participants were included when analyzing exercise class attendance feasibility. Two participants dropped out during the intervention due to disease progression (1) or hospitalization (1). Seven (70%) completed the post-intervention follow-up.

### Participants

The mean age of the 10 ENPAL participants was 64.4 ± 10.3 years. A complete overview of participant demographics, including diagnosis and treatment details, is presented in Table 1.

**Table 1:** Baseline participant demographics

*n = 10*	Range	Mean (SD)	
**Age**	51–83	64.4 (10.3)	
	**Category**	**n**	**%**
**Sex**	Female	6	60
	Male	4	40
**Income** **(thousands)**	< 20	1	10
	40–59.9	2	20
	60–79.9	1	10
	> 80	5	50
	Not specified	1	10
**Education**	Some high school	1	10
	Completed high school	1	10
	Some university/college	3	30
	Completed university/college	3	30
	Some graduate school	2	20
**Marital Status**	Married/common Law	8	80
	Divorced/separated	1	10
	Widowed	1	10
**Employment Status**	Retired	6	60
	Disability/sick leave	4	40
**Cancer Staging**	Stage IV	10	100
**Tumour Marker**	PD-L1 High	6	60
	EGFR activating mutation +	5	50
	ALK translocation +	1	10
**Treatment**	Tyrosine Kinase Inhibitor	6	60
	Immune Checkpoint Inhibitor	2	20
	Immune Checkpoint Inhibitor + Chemotherapy	1	10
	None	1	10
**Treatment Duration**	0–3 months	5	50
	3–6 months	2	20
	> 6 months	2	20
	N/A	1	10

Income listed in Canadian dollars. Treatment duration is calculated from treatment start date to pre-intervention assessment date. *PD-L1* Programmed-death ligand 1, *EGFR*, Epidermal Growth Factor receptor, *ALK* Anaplastic lymphoma kinase

Assessment completion was 85% (10/10 pre-intervention, 7/10 post-intervention). Four of seven post-intervention assessments were completed remotely due to the COVID-19 university closure, limiting collection to PROs and dietary recall only.

Exercise class attendance was 75% (75/100 possible exercise classes available, Table S1, Additional File 2). The most common reasons for missed classes were treatment side effects (7), illness (7), and travel (5). Nutrition and palliative consult attendance was 89% (8/9 attended at least one nutrition and one palliative session). One participant received a second phone-based follow-up nutrition consult. Weekly PA journals were submitted by 78% (7/9) of participants. There were no reported adverse events related to the intervention.

### PROs

#### Physical activity

Self-reported PA, presented in Table 2, did not change significantly throughout the intervention. Participant LSI moderate-strenuous (MS) was 5.3 ± 11.9 pre-intervention and 13.6 ± 17.1 post-intervention (*p* = 0.260). Aerobic MS PA minutes were 8.0 ± 14.4 pre-intervention and 61.4 ± 88.4 post-intervention (*p* = 0.112).

**Table 2:** Pre- and post-intervention PROs

*n = 7*	Pre Mean (SD)	Post Mean (SD)	p-value
**GLTEQ LSI MS**	5.3 (11.9)	13.6 (17.1)	0.260
**GLTEQ LSI MMS**	13.4 (13.7)	19.6 (17.3)	0.492
**Aerobic MS (min/week)**	8.0 (14.4)	61.4 (88.4)	0.112
**Aerobic MMS (min/week)**	68.1 (63.2)	103.6 (103.6)	0.507
**Resistance PA (min/week)**	12.9 (23.6)	21.4 (26.9)	0.505
**ESAS Total**	20.9 (6.5)	19.6 (20.8)	0.854
**ESAS Pain**	2.7 (3.1)	1.7 (2.2)	0.086
**ESAS Tiredness**	4.7 (2.0)	2.9 (2.7)	0.015^*a*^
**ESAS Drowsiness**	1.4 (2.0)	1.0 (1.8)	0.200
**ESAS Nausea**	0.4 (1.1)	0.3 (0.5)	0.788
**ESAS Appetite**	0.7 (1.0)	1.7 (3.0)	0.455
**ESAS Shortness of Breath**	2.1 (1.9)	2.6 (3.3)	0.740
**ESAS Depression**	3.0 (2.4)	2.1 (3.5)	0.634
**ESAS Anxiety**	1.4 (1.7)	2.1 (3.6)	0.588
**ESAS Overall wellbeing**	3.4 (2.1)	2.9 (2.7)	0.654
**FACIT-F**	33.3 (8.2)	36.6 (15.1)	0.429
**FACT-L Total**	100.5 (11.5)	103.8 (23.5)	0.736

All outcomes are presented using mean and standard deviation in brackets. *LSI* Leisure score index. *MS* moderate-strenuous. *MMS* mild-moderate-strenuous. *a* = *p* < 0.05.

PA journals (*n* = 49) indicated a mean weekly PA frequency of 7.26 ± 2.20 (sessions), intensity of 3.63 ± 0.99 (RPE 1–10), and total duration of 262 ± 79 min (Table S2, Additional File 2). Daily life PA (e.g. housework) made up 45.3% of PA recorded, with average weekly frequency of 3.3 ± 1.6 sessions.

#### Symptom burden, fatigue, and quality of life - pre/post intervention

As shown in Table 2, no statistically significant changes were observed for total symptom burden (ESAS total, *p* = 0.854), fatigue (FACIT-F, *p* = 0.429), and QOL (FACT-L total, *p* = 0.736) across the intervention. Individually, ESAS tiredness decreased significantly from pre to post intervention (− 1.86 ± 1.46/10, *p* = 0.015).

#### Symptom burden, fatigue, and energy - pre/post class

As described in the methods, the average pre and post exercise class ESAS symptoms were collected to measure acute changes over the period of an exercise class. Table 3 indicates that exercise classes led to statistically significant acute reductions in ESAS symptom burden (8.2/100 ± 8.7 to 4.7/100 ± 6.0, *p* < 0.001). ESAS pain, tiredness, drowsiness, shortness of breath, depression, anxiety, and overall well-being all improved acutely after an exercise class (*p* < 0.05, Table 3). Fatigue and energy, as measured using single-item thermometers were significantly improved over the duration of one class (Fatigue: 2.4/10 ± 2.3 to 1.4/10 ± 1.4, *p* < 0.001; Energy: 5.7/10 ± 3.1 to 7.0/10 ± 2.7, *p* < 0.001).

**Table 3:** Pre- and post-exercise class PROs

*n = 74*	Pre Mean (SD)	Post Mean (SD)	p-value
**ESAS Total**	8.2 (8.7)	4.7 (6.0)	< 0.001^*a*^
**ESAS Pain**	1.1 (1.8)	0.6 (1.2)	0.001 ^*a*^
**ESAS Tiredness**	2.4 (2.5)	1.6 (2.5)	0.004 ^*a*^
**ESAS Drowsiness**	0.7 (1.4)	0.4 (1.0)	0.002 ^*a*^
**ESAS Nausea**	0.2 (1.0)	0.1 (0.5)	0.095
**ESAS Appetite**	0.8 (1.9)	0.7 (1.8)	0.346
**ESAS Shortness of Breath**	0.8 (1.3)	0.6 (0.8)	0.023 ^*a*^
**ESAS Depression**	0.9 (1.7)	0.3 (0.8)	< 0.001 ^*a*^
**ESAS Anxiety**	0.5 (1.3)	0.1 (0.3)	0.002 ^*a*^
**ESAS Overall wellbeing**	1.5 (1.9)	1.0 (1.6)	0.009 ^*a*^
**Fatigue**	2.4 (2.3)	1.4 (1.4)	< 0.001 ^*a*^
**Energy**	5.7 (3.1)	7.0 (2.7)	< 0.001 ^*a*^

All outcomes are presented using mean and standard deviation in brackets based on 74 exercise classes (one missing form) across nine participants. *a* = *p* < 0.05.

#### Dietary recall

Mean daily intake was 20.6 ± 3.4 kcal/kg and 0.79 ± 0.18 g/kg of protein before starting ENPAL (Table 4). Post-intervention daily intake was 23.9 ± 7.4 kcal/kg and 1.1 ± 0.49 g/kg of protein. No statistically significant dietary changes were observed after completion of ENPAL (Table 4). All participants confirmed that the dietary recall represented a typical day for them.

**Table 4:** Pre- and post- intervention dietary patterns based on ASA24-hour dietary recall

*n = 7*	Pre Mean (SD)	Post Mean (SD)	p-value
**Calories (kcal)**	1513 (398)	1755 (655)	0.386
**Calories (kcal/kg)**	20.6 (3.4)	23.9 (7.4)	0.412
**Protein (g)**	57.7 (19.1)	79.5 (33.7)	0.167
**Protein (g/kg)**	0.79 (0.18)	1.1 (0.49)	0.150
**Fat (g)**	58.7 (22.9)	78.6 (39.1)	0.154
**Fat (g/kg)**	0.78 (0.25)	1.0 (0.34)	0.182
**Carbohydrates (g)**	180.5 (55.5)	170.1 (51.2)	0.732
**Carbohydrates (g/kg)**	2.5 (0.78)	2,4 (1.0)	0.835

All outcomes are presented using mean and standard deviation in brackets. *a* = *p* < 0.05.

### Physical function

Physical function was assessed for all participants pre-intervention and three participants post-intervention, as shown in Table S3, Additional File 2. Due to the COVID-19-related university closure, physical function measures could not be collected for the remaining participants post-intervention. No statistical tests were performed due to limited data.

### Nutrition and palliative symptom management consults

An overview of the information provided during nutrition consults is presented in Table S4, Additional File 2. Increases in energy intake (62.5%), protein (100%), and fat (50%) were commonly recommended. Dietitians suggested solutions such as the use of nutrient dense supplements (62.5%) and unflavored protein powder (87.5%). Management of nutrition-related symptoms included diarrhea (37.5%), poor appetite (50%), and dry mouth (37.5%).

Palliative symptom management consults are summarized in Table S5, Additional File 2. Pain and dyspnea were discussed in 75% of consults. Discussions around mood, fatigue, goals of care, insomnia, and constipation featured in 62.5% of consults. Two common interventions presented by physicians were analgesics (62.5%) and other medications such as laxatives and bowel medications (37.5%).

### Qualitative perspectives

Three themes emerged from participant interviews: 1) a multimodal program is feasible, 2) the value of the multimodal program, and 3) how to improve the multimodal program. Additional participant quotes are presented in Table S6, Additional File 2.

#### Theme 1: a multimodal program is feasible

Participants described the completion of pre−/post- class questionnaires and pre−/post- study assessments to be reasonable and helpful, providing a benchmark for physical and psychosocial outcomes during treatment that they could refer to throughout the program.

*That’s what got me definitely hooked onto the program, was the assessment. It was very simple to fill out all the questions and I had no problems whatsoever.* ***P5.***

A satisfaction with the frequency, duration, time, and type of exercise classes and home-based exercise prescriptions in ENPAL was noted. Exercise progression and variation was viewed as acceptable, although one participant would have preferred more variety and a quicker progression.

*The timing is excellent. It’s not too long and it’s not too short. We all enjoy, and I’m not watching and looking at the clock like oh, I need to go home, no, I’m never do that, I enjoy 100% doing the exercise.* ***P2.***

*Just too routine, for me. Same thing over and over.* ***P1****.*

The importance of an exercise professional was highlighted for fostering a feasible and successful program, allowing participants to feel safe and supported during exercise and motivated to attend classes.

*They [the instructor] were really marvelous. […] I think they are a great part of having the successful program like that […] it’s the trainers. The way the kind of aesthetic they invoke on the setting, and also their expertise played an important part.* ***P8.***

The nutrition and palliative consults were perceived as acceptable. Participants found them helpful to address unique nutritional needs (e.g. increasing protein intake during cancer treatment), while appreciating the expertise of the palliative care physicians. The palliative symptom management consult was less suitable to those with limited pain or side effects. .

*To be aware that he’s [the Doctor] there, and we had a good chat and that was all, there was nothing directly relevant because I don’t have any pain […] we established that there is such a thing as the pain clinic, that there is real serious expertise involved, that if I get to that situation that I can expect some skill.* ***P4.***

#### Theme 2: the value of a multimodal program

Participants noted the physical and psychosocial benefits of the ENPAL program for improving QOL, bringing normalcy back to their lives, increasing energy, decreasing fatigue and anxiety, improving sleep and physical function, and enhancing their outlook on life.

*So overall, I’m really happy that I’ve been a part of it, it’s been good for me, it’s caused my quality of life to be better […].* ***P4.***

*Well, I think it’s definitely the thing that like it helped to get you back to feeling your normal self again. And physical activity. And […] cancer is, is in many cases, in my case, it’s not a treatable thing. So, it’s just that, at least for some time, the goal here is to enjoy life as it is, as normal as it as I possibly can.* ***P8.***

ENPAL was perceived as beneficial for increasing confidence and motivation for engaging in PA, encouraging behavior change, enabling participants to integrate PA into their cancer journeys and lifestyles. Central to this was the participants’ improved self-efficacy for activities of daily living.

*I wasn’t sure even I can, do the exercises […].Especially I just came out of the hospital and you know it was downhill completely. So, the program actually helped me to get back to the normal and realize that I can still, with the medication that I’m taking, get my energy level up and, get my muscles to move again and actually, […] I think it somehow made me realize that exercise is a better, a good treatment to the side effects that I have from that medication that is primarily the muscle soreness that I have.* ***P8.***

The social support from fellow participants and instructors within the group-based exercise classes was widely appreciated, improving willingness to attend and contributing to psychosocial benefits (e.g. mood, QOL).

*So, we all know we have the end stage of cancer and like we will think about what happened to my friend, what happened to them why they are not coming. So, I think it’s a social life, I got some social life back […] I just feel it’s so important to have a group of people to work together, and we are on the same boat.* ***P2.***

#### Theme 3: how to improve a multimodal program

Participants provided suggestions to improve ENPAL moving forward with additional online resources for exercise and nutrition, one-on-one sessions with other experts (e.g. psychologist), and ongoing support during and after the program, especially for at-home PA.

*I know there’s apps that you can get where they have exercises online, but it would be nice to have the one specific for you online […] you know, just in case you forget when you go home.****P10.***

*I would like to see them introduce and have a [one-on-one] counsellor in there [in ENPAL]. I think talking about the cancer and about their feelings, their emotions.* ***P5.***

*Doing them [the exercises] at home, I wasn’t as motivated. As opposed to being at the university, I loved the outing and I loved getting out. That’s what I looked forward to. […] So I found that very difficult doing them at home.* ***P5.***

## Discussion

As the number of ALC patients continues to grow, innovative interventions that address multiple components of QOL are necessary to combat morbidity among these patients [1–3]. While PA, nutrition, and symptom management interventions have been shown to enhance QOL independently, previous research in cancer populations suggests that integrated multimodal approaches may be better suited to holistically address QOL needs [5–8, 27]. To date, only bimodal interventions (combining two of PA, nutrition, and palliative symptom management) have been studied in advanced cancer populations, which may not address all physical and psychological patient needs. Therefore, the current study was designed to evaluate the feasibility of a novel and comprehensive wellness intervention that combined PA, nutrition and palliative care consultations. Given the burden of disease, morbidity, and appointments burden among ALC patients, determining feasibility was an important first step to inform future research on multimodal supportive care for this population.

### Feasibility

Our results indicate that the novel multimodal PA, nutrition, and palliative symptom management intervention was safe, feasible, and well-tolerated. Recruitment (44%), attendance (75%), and assessment completion (85%) rates exceeded the pre-determined feasibility values (30, 60, and 70%, respectively). No adverse events related to the intervention were reported and interviews indicated high participant satisfaction.

A 44% recruitment rate is comparable to previous exercise interventions in advanced cancer, where the mean rate was 49% [28]. A factor that positively impacted our recruitment rate was the strong support and direct referral from oncologists. The research coordinator met with the oncologists to discuss the trial and how PA would be modified to meet patient needs and attended clinic to help the health care team determine patient eligibility. Interestingly, the most common reason for ineligibility in this study was non-English speaking (15 patients), emphasizing the need for translation of intervention content to ensure equitable access to supportive care.

Intervention (70%) and assessment (85%) completion was comparable to the 76% in past PA interventions for advanced cancer [28]. As with previous interventions, disease progression was the most common reason for attrition in ENPAL (66%, 2/3 of participants who did not complete the intervention) [28]. Completion rates of previous bimodal nutrition and PA interventions in advanced cancer ranged from 42 to 70%, indicating that the addition of a palliative component in the ENPAL program likely did not affect study completion [8].

Qualitative interviews provided a deeper understanding of recruitment, assessment, and intervention feasibility. Participants reported that in-person interaction with the study team was important to help them overcome doubts about their ability to participate. Prior qualitative work in advanced cancer suggests that although perceptions of PA are positive, social support, such as that provided by the study team on initial contact, may be key to motivating health behavior change [29]. In addition, the assessments were not only feasible but perceived as valuable to help participants understand their current condition and progress. Therefore, the personal support provided throughout recruitment and the assessment reports provided to participants both proved crucial to a positive intervention experience.

### A multimodal intervention - physical activity

The PA prescription was individualized based on prior PA experience, current physical function, and current clinical condition, with weekly recommendations aimed at progressing towards ACSM’s Cancer Exercise guidelines (i.e. weekly: 90 min of moderate intensity aerobic training, 2 days of resistance training, and flexibility training on most days) [14]. One of the PA sessions was done in the in-person group class setting. Participants recorded an average of once weekly cardiovascular (1.18 ± 1.50) and resistance training (1.34 ± 1.52), as well as some stretching, as prescribed (Table S2, Additional File 2). In-person class attendance was 75% in ENPAL, which was very high compared to 44–45% in previous trials with ALC patients [30, 31]. This may be related to class frequency, which was two or three times weekly within previous studies, compared to once weekly in ENPAL, suggesting that the latter may be more suitable for this population [30, 31]. Taken together, PA class attendance and PA logs indicate that following the individualized progressive PA prescription was feasible, although self-report PA levels suggest that not all ENPAL participants met ACSM Cancer exercise guidelines post-intervention.

ENPAL classes were offered on 2 days per week, allowing participants to attend either or both. Participants appreciated this flexibility and the ability to individualize exercise frequency, as noted during post-intervention interviews. This likely contributed to increased attendance rates. Perspectives on the recommended PA frequency (i.e. once weekly in-person group class and 2 or more days per week individual PA at home) varied, with the frequency being described as more than enough or just right, whereas some participants preferred twice weekly in-person classes. Therefore, individualization is key when prescribing PA for ALC patients. An additional facilitator for attendance was enjoyment, which has previously been reported as a key stimulus for exercise in older adults, with strong positive associations to exercise persistence [32, 33]. Factors that contributed to enjoyment were social support from instructors and peers, as well as the suitability of the exercises. As class times were tailored to participants, only 8% (2/25) of missed sessions in the ENPAL program were due to conflicting appointments, compared to 54% in a similar intervention for advanced cancer [34].

Despite the tailored program, participants expressed their struggles with at-home exercise due to lack of motivation and social support. Previous qualitative research on exercise in ALC noted similar barriers [33]. Behavior change education in ENPAL was designed to support adoption and adherence to at home PA, including eliciting social support from family and friends, but participants still reported lack of motivation. Additional supportive resources (e.g. health coaching, technology-based support such as live or pre-recorded exercise videos), as suggested by participants during interviews, may be required to overcome persistent barriers.

No adverse events were noted in relation to the intervention, suggesting that, in the presence of professional tailoring and monitoring, ALC populations can exercise safely and should not be excluded from general cancer-exercise programs, as long as the exercise prescription is tailored to meet participant needs. In line with prior qualitative work on exercise in ALC, ENPAL participants preferred group-based to at-home exercise due to the enhanced motivation and social support [35]. However, some participants may need further support to motivate at-home PA and reach recommended PA levels.

### A multimodal intervention - palliative symptom management

The present work is the first to integrate palliative care consultations, which has demonstrated potential for improving QOL, into a multimodal intervention for ALC [5, 6, 36]. The palliative care consult was feasible, as evidenced by 89% attendance and positive feedback in interviews (Table S5, Additional File 2). Interestingly, participants noted that some topics (e.g., pain) were not relevant to them, yet patient-reported pain decreased by more than 5% over the intervention, which was clinically meaningful (Table 2) [37]. Previous work, where patients chose symptom management topics of interest, resulted in enhanced QOL, echoing ENPAL participant requests for a more personalized approach [36]. Further research is warranted to evaluate palliative symptom management in multimodal interventions.

### A multimodal intervention - nutritional counseling

The feasibility of the nutrition consults in ENPAL was demonstrated by 89% attendance and positive participant perspectives, adding to the evidence for feasibility of nutritional interventions in advanced cancer and lung cancer [8, 11, 38–40]. As with the palliative care component of the intervention, all participants attended an initial dietary counselling session, with the option to attend additional sessions. One participant received a second phone-based follow-up counselling session. While feasible, the reduced intervention intensity in ENPAL, compared to more frequent consults or specific nutritional prescriptions in previous research, may limit its impact on nutritional outcomes [8, 11, 38–40]. Practical advice to increase protein and overall energy intake was not statistically reflected in the reported post-intervention dietary measures, although this may be due to the limited power of this feasibility study to detect changes in secondary nutritional outcomes. As with PA, maintenance of BMI and nutritional intake is promising, given that weight maintenance is a predictor for improved prognosis in lung cancer and BMI can decrease rapidly in ALC patients [41]. Larger, multi-arm, controlled trials are required to assess the effects of nutrition consults in a multimodal intervention on QOL and nutritional outcomes.

### Preliminary examination of PROs

Secondary to determining feasibility, a preliminary examination of intervention effects on PROs was performed to inform future trials. As found in previous PA interventions, participants did not report significant changes in PA, indicating that the intervention may have supported PA maintenance, counteracting usual declines in PA among advanced cancer populations [42–45]. Due to prevalent dyspnea, pain, fatigue, psychosocial challenges, and demanding treatments, PA adherence can be difficult for individuals living with ALC, which may have also impacted increased PA levels [2, 3]. However, an adequately powered controlled trial is required to assess the potential impact of this multimodal intervention on PA behavior.

As with PA, QOL can decrease rapidly in ALC patients due to treatments, disease progression, and worsening comorbidities [46]. However, ENPAL participants did not report significant declines in overall wellbeing or QOL across the intervention period (Table 2). Similar to past feasibility studies of PA interventions in ALC, QOL, symptom burden, and general fatigue were maintained [44, 47]. The intervention may have contributed to clinically significant decreases in tiredness and pain (> 5%, although only tiredness was statistically significant, *p* = 0.015), which is promising [35]. Larger trials are needed to investigate potential effects on QOL and the specific impact of each component of this multimodal approach [14, 48, 49]. The low initial symptom burden and high QOL of our sample at baseline, along with completion of PROs during the COVID-19 pandemic (4/7 participants) remain as uncontrolled confounders when examining intervention effects on QOL. Finally, the current study provides preliminary evidence for the acute benefits of an exercise class on symptom management in ALC patients. Statistically significant and clinically meaningful (i.e. > 5%) acute post-class improvements in fatigue, energy, tiredness, depression, pain, and wellbeing were observed that mimic findings from an exercise intervention in head and neck cancer (Table 3) [37, 50]. Future work is recommended to explain these acute changes, as they have potential to enhance long-term PA adherence [51].

Qualitative data highlighted the potential QOL and health behaviour benefits of the multimodal intervention. Participants noted improvements to physical (e.g. physical function, energy) and psychosocial (e.g. normalcy, anxiety, positivity) well-being, as well as improved self-efficacy and social support. For example, one participant stated, “So overall, I’m really happy that I’ve been a part of it, it’s been good for me, it’s caused my quality of life to be better.” Previous qualitative work in advanced cancer links these benefits to positive attitudes towards PA and increased motivation for PA [29]. Therefore, individual benefits may initiate a positive feedback loop, leading to further improvements in well-being.

### Strengths, limitations, and future directions

The in-clinic recruitment process and tailored intervention delivery were both key strengths that contributed to the success of the ENPAL program. Involvement of clinical staff in the study design prompted clinician buy-in, while rolling recruitment allowed participants to receive supportive care immediately, which is crucial for advanced cancer populations. Furthermore, social support was facilitated by instructors and the group-exercise environment, potentially supporting class attendance. Finally, individualization and safety were highlighted using small class sizes and individualized PA programs. Qualitative data collected indicates intervention feasibility and value, which has not been commonly included in PA interventions for ALC [47, 52].

There are several limitations to the current feasibility pilot that can be addressed using a larger controlled trial. As this was a feasibility trial, the study did not include a control group, and therefore we were unable to draw cause and effect conclusions. It was also not powered to detect differences over time. Therefore, the results must be cautiously interpreted. Furthermore, statistically significant changes, indicating potential benefits of ENPAL, may represent false positives due to lack of correction for multiple comparisons. Due to voluntary recruitment, highly motivated patients were more likely to participate in ENPAL, therefore limiting the generalizability of the results. Additionally, without objective PA monitoring, PA outcomes remain prone to self-report biases. As participants reported relatively low symptom burden, high QOL, well-preserved exercise capacity, and moderate PA at baseline, results may not be generalizable to all advanced lung cancer patients. As the ALC population remains understudied, comparisons to previous work must be interpreted with caution given that included populations likely experienced different side effects (i.e. type and severity) due to different treatments. Finally, although a variety of lung cancer subtypes and treatment regiments were represented, additional research is needed to understand differences in capacity for and response to multimodal interventions depending on demographic characteristics and treatment differences.

## Conclusion

In conclusion, the present study indicates that a multimodal exercise, nutrition, and palliative symptom management intervention is safe and feasible in patients with advanced NSCLC. One participant concluded, “it somehow made me realize that exercise is a better, is a good treatment to the side effects that I have from that medication.” The potential benefits of a multimodal intervention to improve QOL in ALC, including its positive effects on PROs and acute improvements in symptom burden after the exercise class, warrant further research.

## Supplementary Information

**Additional file 1.** Sample exercise program

**Additional file 2.** Supplementary tables

## Data Availability

The datasets generated and/or analysed during the current study are not publicly available as they contain information that may compromise participant privacy/consent but are available from the corresponding author (SNCR) on reasonable request.

## Abbreviations

QOL: Quality of life
ALC: Advanced lung cancer
PA: Physical activity
RCT: Randomized controlled trial
NSCLC: Non-small cell lung cancer
ENPAL: Exercise, Nutrition, and Palliative care in Advanced Lung cancer
ECOG: Eastern Cooperative Oncology Group
ESAS: Edmonton Symptom Assessment System
HREBA.CC: Health Research Ethics Board of Alberta – Cancer Committee
PROs: Patient-reported outcomes
GLTEQ: Godin leisure-time exercise questionnaire
MVPA: Moderate-vigorous physical activity
LSI: Leisure score index
FACT-L: Functional assessment of cancer therapy – Lung
FACIT-F: Functional assessment of chronic illness therapy – Fatigue
PWB: Physical well-being
SWB: Social/family well-being
EWB: Emotional well-being
FWB: Functional well-being
LCS: Lung cancer subscale
ASA: Automated self-administered
CPFLA: Canadian Physical Activity Fitness and Lifestyle Approach
BMI: Body-mass index
SD: Standard deviation
PD-L1: Programmed-death Ligand 1
EGFR: Epidermal growth factor receptor
ALK: Anaplastic lymphoma kinase
MET: Metabolic equivalent of task
MS: Moderate-severe
MMS: Mild-moderate-severe
