# Supplementary material for: Feasibility of a multimodal exercise, nutrition, and palliative care intervention in advanced lung cancer
Source: BMC Cancer. 2021 Feb 13;21:159. doi: 10.1186/s12885-021-07872-y (PMC7881342; doi:10.1186/s12885-021-07872-y)
Supplement: Supplementary file 1 — Additional file 1. Sample exercise program [file 12885_2021_7872_MOESM1_ESM.docx]

**THE FOLLOWING IS AN EXAMPLE OF THE INSTRUCTIONS AND A SAMPLE EXERCISE PROGRAM USED IN THE ENPAL STUDY. ALL EXERCISE PROGRAMS WERE TAILORED TO MEET THE PARTICIPANT’S UNIQUE NEEDS AND ABILITIES, AND THEREFORE VARIED FROM THIS SAMPLE. ALL IMAGES HAVE BEEN REMOVED FROM THE PUBLISHED PROGRAM FOR COPYRIGHT/PRIVACY REASONS**

**Instructions**

DEAR [Insert participant name]

Welcome to the EXERCISE, NUTRITION AND PALLIATIVE CARE in ADVANCED LUNG CANCER Study (ENPAL). Included in this package, you will find your home exercise program. You will learn these exercises with your ENPAL Study Exercise Specialist, and in your ENPAL group classes.

The purpose of this exercise program is to provide guidance for your tailored workout, where you are in control. The goal of staying active and following this program is to increase your daily energy levels and decrease fatigue. Therefore, it is important to listen to your body and choose how many exercises to do depending on your level of energy.

You will start with one complete exercise program. We will focus on these key exercises each week and continue to add more as appropriate. Please start each workout with a light warm up and end each workout with a cool down stretch while your muscles are still warm. Our goal is for the workouts to be short enough to fit into your daily routine, for example 15-30 minutes each. Your individualized program detailing how many days per week you will do your exercises will be listed below on the second page of this exercise prescription form.

The purpose of a short warm up is to warm-up your muscles. The number of times you do each exercise depends on your fatigue and energy levels, and should also be guided based upon how you feel during exercise, using your rate of perceived exertion (RPE) after each completed circuit. The fatigue thermometer can be used to help you asses your fatigue.

Over the weeks, we may add different exercises based on your energy levels and ability. These new exercises will be broken into different exercise programs, based on the traffic light system. The traffic light system includes intensities from light (red), medium (yellow), and hard (green). The red exercise program should be utilized on a day of high fatigue and low energy. The yellow exercise program should be utilized on a day of moderate energy levels. The green exercise program can be used if you have lower fatigue and moderate to high energy and you’re looking for a challenge. Again, for now we will focus on using one program.

This is a home training program, therefore all exercises are able to be performed either at home or a local gym. Exercise bands, an exercise ball, and dumb bells are helpful for the program. We have provided you with an exercise ball and an exercise band.

The program can also be used on a mobile device by downloading the Adobe Acrobat Reader on your smartphone, which is a free App that can be found in the App Store (for Apple products) or Google Play (for android products). After the download, the document can be opened in the app.

**WEEKLY EXERCISE PROGRAM**

Your goal over the next 4-weeks is to work towards 2-3 exercise sessions per week. By the end of the 12-week program, we may increase this based on how you tolerate this initial program. Your ENPAL Study Exercise Specialist will help you to modify this so it suits your needs.

**YOUR PERSONALIZED EXERCISE PLAN**

**AEROBIC EXERCISE:** 10-15 minute moderate intensity aerobic exercise sessions (i.e. walking) 2-3 times per week. You will get one of these sessions in your group exercise class!

Prescribed by my Exercise Specialist:

| Frequency | 2-3/10 |
| --- | --- |
| Intensity | Light to moderate |
| Duration | 5-7 minutes |
| Activates I enjoy (i.e. walking) | [insert activities the participant identified] |

**RESISTANCE EXERCISE:**

1-3 sessions of resistance exercise per week. You will get one of these sessions in your group exercise class!

| Frequency | 1-3 (one in your group exercise session, 1-2 at home) |
| --- | --- |
| Intensity | Light to moderate |
| Duration | 20-30 minutes |
| Activates | Home exercise program |

**FLEXIBILITY EXERCISE:** Try to include stretching exercises most days of the week! See your home exercise program for examples. You will also get one of these sessions in your group exercise class!

For any concerns or questions contact: [insert study staff and class instructor contact here].

Estimated Time for Workout: 10-20 min

| Exercise | Repetition | Description | Alternatives |
| --- | --- | --- | --- |
| **Sit to Stand** | 45 seconds or  8-12 reps | Sit on a chair that has been placed against a wall to prevent slipping. With your feet slightly apart and your hips at the edge of the seat, lift your hips up from the seat to stand. Slowly return to sitting.  Focus on having an engaged core (either interlace your fingers and reach forward or cross your arms to the opposite shoulder in front of your chest). | -Seated leg extension -Ball Squats |
|  | You should feel this in your:  Front Thigh  (Quads) |  |  |
| **Wall Angels** | 45 seconds  or  8-12 reps | Stand up with your back and head against the wall, with the head, shoulder blades and bum in contact with the wall. Tighten your abdominal muscles, gently pull your shoulder blades toward each other and the back of your hands down into the wall. Without letting your shoulders roll forward, slowly slide your arms on the wall to raise them overhead—as high as possible with the good posture and without pain. Stop the movement when something pops off the wall – i.e. arm comes off the wall. Do not let your shoulders shrug toward your ears.  Stay in a comfortable range of motion. | -Snow Angels  - I-Y-T’s |
|  | You should feel this in your:  Shoulders |  |  |

Recommended Repetitions of circuit: 1-3 (depending on rating of fatigue and rating of energy)
Intensity: Light (RPE 1-3)

| Exercise | Repetition | Description | Alternatives |
| --- | --- | --- | --- |
| **Walking around** | 45 seconds | Walk around in the house potentially walk stairs or change speed to increase intensity. | -Marching  -Jogging on the spot  -2in 2out |
| **Wall Push Ups** | 45 seconds  or  12-15 reps | Stand about 12 to 18 inches away from a wall while facing it and place your hands on the wall at shoulder level. Keeping your shoulders down and back, gradually bend your elbows to bring your face and forearms to the wall.  Return to the initial position and repeat. | -Band press |
|  | You should feel this in your:  Chest |  |  |

| Exercise | Repetition | Description | Alternatives |
| --- | --- | --- | --- |
| **Tandem walk** | 45 seconds | Challenge balance by walking with a tandem gait, one foot directly in front of the other. Turn 180 degrees and return back using the same gait.  Difficulty levels:   1. Focus on point on the floor 2. Set your gaze straight ahead 3. Close your eyes | -Treepose  -On two legs eyes closed  -Single leg balance |
| **Standing Row** | 45 seconds or  8-12 reps | Anchor a resistance band in front of you at chest level and hold the ends in your hands.  Pull the band with your elbows near your sides and keep your chest out and shoulders down and back.  Do not move you head forward as you pull. | -Seated row  -One arm row  -Reverse Fly |
|  | You should feel this in your:  Back (Lats) |  |  |

| Exercise | Repetition | Description | Alternatives |
| --- | --- | --- | --- |
| **Birddog** | 45 seconds | Get on your hands and knees (four-point position) with your knees and hands, hip and shoulders width apart. Your back is in neutral position (slightly arched) and your chin must be tucked in. Activate your lower back by bringing your belly button inward and by activating your pelvic floor muscles. Maintain a steady abdominal breathing while you simultaneously lift one leg backwards and the opposite arm overhead keeping your back in neutral position. Return to the initial position and repeat with the other leg and arm.  Levels of difficulty:   1. One limb at a time 2. Opposite arm and leg 3. On an exercise ball, lift opposite arm & leg | -Glute Bridge  -Clamshells  -Fire Hydrants |
|  | You should feel this in your:  Core/ Back/ Glute |  |  |
| **Clamshells** | 45 seconds  or  8 reps each side | Lie on your side with both legs slightly bent. Lift your top leg, keeping your pelvis stable, your leg slightly bent and your heels together. Rotate your hip so that your foot and your kneecap are pointing upward during the movement.  Return to the initial position and repeat.  The difficulty can be increased by adding a resistance band just above your knee. | -Glute Bridge  -Birddog  -Fire Hydrants |
|  | You should feel this in your:  Side of Hip  (Glute Med) |  |  |

| Exercise | Repetition | Description | Alternatives |
| --- | --- | --- | --- |
| I – Y – T | 45 seconds | Lay on your back on the floor. Your arms stay extended throughout the exercise. First form an “I” with your arms then a “Y” and finally a “T”. Repeatedly form these letters. Start slowly and avoid ranges of motion, which cause pain. Very similar to the YMCA dance.  Levels of difficulty:   1. On the floor 2. Elevated (as shown in the picture) | -Wall Angels  -Snow Angels |

| **Glute Bridge** | 45 seconds  or  12-15 reps | Start laying on your back with knee bent at 90°. Squeeze the glutes and lift the hips off the ground.  Feet stay flat on the ground.  Ideal position is if the body is in one line from chest to the knees. Do not go too far in the range of motion. Repeat without touching the ground. | -Birddog  -Clamshells  -Fire hydrants |
| --- | --- | --- | --- |
|  | You should feel this in your:  Core/ Buttocks (Glute) |  |  |

| **Stretch Options**  Please warm up with light aerobic activity for 5 to 15 minutes prior to your program. |
| --- |

|  |  | **Snow angel** ► 1-3 sets 10 reps 3-5 sec | |
| --- | --- | --- | --- |
|  |  |  | While lying on back with knees ben start with your arms straight out to the side and slowly raise them up and over your head. You can have a slight bend in the elbows as your range of motion allows. |
|  |  | **Foam Roller - I, Y and T** ► 1-3 sets 10 reps 5-10 sec (Can be done without foam roller) | |
|  |  |  | • I’s: Sit on one end of the roller and lay back so that your head is supported on the roller, and your spine is in line with the roller. Bend knees to provide balance. Bring your arms straight up over your head, leading with your thumbs and allow gravity to cause a stretch.  • Y’s: Repeat above exercise but change arm position so that you look like a Y. Palms facing up  • T’s: Repeat above exercise but change arm position to straight out at your sides (so that your body is in the shape of a T) |
|  |  | **Stretch - Low back and hips** ► 1-3 sets 20-30 sec | |
|  |  |  | Lie on your back with your knees bent and feet flat on the floor. Keep arms at sides to help stabilize the upper body. Keeping knees together and gently let your knees fall out to each side until a stretch is felt in the hip and/or low back. Hold for 10-30 seconds and repeat on the other side. Repeat this sequence 5-10 times on each side. |
|  |  | **Stretch - Double Knee to Chest** ► 1-3 sets 20-30 sec | |
|  |  |  | Lying on your back, bring both knees towards your chest. Give your knees a hug to increase the stretch in your low back. You can hold on over the top of the lower leg as in the picture (or under the lower leg to reduce stain through the knee, if felt). Find a comfortable stretch and hold 20-30 seconds. |
|  |  | **Stretch - glute max** ► 1-3 sets 20-30 sec | |
|  |  |  | Lie on your back, grab one leg at the knee and pull towards your chest. Keep your other leg straight, if possible.  Try to keep your lower back in contact with the floor. |
|  |  | **Stretch - Glute med** ► 1-3 sets 20-30 sec | |
|  |  |  | Lay flat on your back, lift one leg so that both the knee and hip are bent to 90 degrees. Grab the knee of the lifted leg with the opposite hand and gently pull the leg across your body until you feel a stretch in the buttock of the lifted leg. Make sure to keep the hip of your straight leg on the ground. |
|  |  | **Stretch - Hamstring** ► 1-3 sets 20-30 sec | |
|  |  |  | Lie on your back and lift one leg up. Keep your other foot on the ground, bending your knee.  Grasp behind your lifted thigh, near the knee, with both hands. Maintain your leg in a straight position while attempting to pull it closer to your chest. Hold for 30 seconds. Relax and repeat on the other leg.  It is very important to keep the lifted leg straight throughout the stretch. |
|  |  | **Stretch - Hip** ► 1-3 sets 20-30 sec | |
|  |  |  | Lie on your back and bend one knee to 90 degrees. Cross the other leg, placing the ankle on the thigh of the bent leg. Grasp around the thigh of the bent leg and pull towards your chest. Hold for 30 seconds. Relax. Repeat 2-3 times on each side. |
|  |  | **Stretch - Quadriceps** ► 1-3 sets 20-30 sec | |
|  |  |  | Lie on your side.  Bring your lower leg up towards your chest to help maintain neutral pelvis.  Grab onto your other ankle, push your hip forward, and pull your foot towards your buttock until you feel a stretch in the front of your thigh.  Stop if you feel pressure in your lower back. Keep your knee in line with your hip. |
|  |  | **Stretch - Side lying arm circles** ► 1-3 sets 10 reps | |
|  |  |  | Start by lying on your side with your knees bent where comfortable and your bottom arm or a pillow supporting your head. With the top arm reaching out as far as you can, make slow circles moving in all directions. In front and above your head, try to keep finger tips brushing along the floor then as you move behind you, your arm will come up but still reach back as much as you can to stretch the chest. Do in both directions (clockwise and counter clockwise).Then repeat on the other side. |
|  |  |  | |
|  |  |  |  |
|  |  | **Cat Camel** ► 1-3 sets 10 reps 3-5 sec | |
|  |  |  | Start on all fours. Slowly curl your upper back so you like an angry cat, while tucking your chin towards your chest. Hold for 2 seconds. Then return to the starting position, allowing your back to arch and look up towards the ceiling. Hold for 2 seconds. Repeat 10 times. |
|  |  | **Stretch - Hip Flexors** ► 1-3 sets 20-30 sec | |
|  |  |  | Start exercise from a kneeling position, bring one leg out in front of you with your knee bent to 90 degrees. Keep your other knee in contact with the mat and your back straight. Gently push your hip forward until you feel a stretch in the front thigh of your back leg. |
|  |  | **Stretch - Pectorals** ► 1-3 sets 20-30 sec | |
|  |  |  | Start with your elbow at shoulder height and bent to 90 degrees. Place forearm and elbow against a wall. Step forward with the leg closest to the wall and lean forward until you feel a stretch in your chest. You may need to turn your body away from the wall. Alternatively, you can stand in a doorway and place your forearms against the door frame. Step forward with one leg and lean your body forward until you feel a stretch in your chest. Move your arm up and down to change where you feel it. |
|  |  | **Stretch - shoulder internal rotation** ► 1-3 sets 20-30 sec | |
|  |  |  | Grasp a towel or strap with one hand and position behind your head. Reach other hand behind you at about the height of your low back and grasp other end of towel or strap. Letting the lower hand/arm relax, gently pull it up the spine, making the other arm do the work. Stop when a gentle stretch is felt in the front of the shoulder of the lower arm. |
|  |  | **Stretch - calf (wall)** ► 1-3 sets 20-30 sec | |
|  |  |  | Stand facing a wall with your hands against the wall for support. Place your forefoot and toes of one foot against the wall, keeping your heel in contact with the floor. Gently lean forward until you feel a stretch in the calf of the leg that is resting against the wall. Hold for 30 seconds. Then bend the front knee until you feel a stretch in the lower part of the calf. Hold this position for 30 seconds. |
|  |  | **Stretch - chin to chest neck stretch** ► 1-3 sets 20-30 sec | |
|  |  |  | With good posture, sitting or standing up tall, gently move your head down towards your chest until a gentle pull is felt up the back of the neck. Hold 20-30 seconds. |
|  |  |  |  |
|  |  | **Stretch - Neck Side Flexion** ► 1-3 sets 20-30 sec | |
|  |  |  | Keeping ears in line with shoulders so that head is not too far forward or backwards. Gently tilt head towards your shoulder until a stretch is felt in the opposite side in the upper trapezius/neck area. Repeat towards the other side. |
