# Supplementary material for: Feasibility of a multimodal exercise, nutrition, and palliative care intervention in advanced lung cancer
Source: BMC Cancer. 2021 Feb 13;21:159. doi: 10.1186/s12885-021-07872-y (PMC7881342; doi:10.1186/s12885-021-07872-y)
Supplement: Supplementary file 2 — Additional file 2. Supplementary tables [file 12885_2021_7872_MOESM2_ESM.docx]

**Feasibility and preliminary efficacy of a multimodal exercise, nutrition, and palliative care intervention in advanced lung cancer**

**Additional file 2: Supplementary Tables**

**Table S1.** ENPAL exercise class attendance breakdown. **Table S2.** Self-reported PA from weekly activity journals. **Table S3.** Pre- and post-intervention physical function measures. **Table S4.** Frequency of information provided to participants during ENPAL nutritional consults. **Table S5.** Frequency of information provided to participants during ENPAL palliative symptom management consults. **Table S6.** Participant quotes from semi-structured interviews.

**Table S1.** ENPAL exercise class attendance breakdown.

|  | **Week** | | | | | | | | | | | | | |
| --- | --- | --- | --- | --- | --- | --- | --- | --- | --- | --- | --- | --- | --- | --- |
| **ID** | **1** | **2** | **3** | **4** | **5** | **6** | **7** | **8** | **9** | **10** | **11** | **12** | **13** | **14** |
| **1** | 1 | 1 | 3 | 3 | 1 | 1 | 1 | 1 | 1 | 1 | 1 | 1 |  |  |
| **2** | 1 | 1 | 1 | 1 | 1 | 2 | 2 | 2 | 3 | 3 | 1 |  |  |  |
| **3** | X | X | X | X | X | X | X | X | X | X | X | X | X | X |
| **4** | 1 | 1 | 1 | 1 | 1 | 1 | 1 | 1 | 1 | 1 | 1 | 1 |  |  |
| **5** | 1 | 1 | 1 | 1 | 1 | 3 | 3 | 3 | 3 | 3 | 3 | 3 | Z | Z |
| **6** | 1 | 1 | 1 | 1 | 3 | 3 | 3 | 1 | 1 | 3 | 3 | 3 | 3 | 3 |
| **7** | 1 | Y | Y | Y | Y | Y | Y | Y | Y | Y | Y | Y | Y | Y |
| **8** | 1 | 1 | 1 | 1 | 1 | 3 | 1 | 1 | 1 | 3 | 3 | 3 | Z | Z |
| **9** | 1 | 2 | 1 | 1 | 2 | 2 | 2 | 1 |  |  |  |  |  |  |
| **10** | 1 | 1 | 1 | 1 | 1 | 3 | 3 | 1 | 1 | 1 | 1 | Z | Z | Z |

1: attended. 2: attended 2 sessions. 3: did not attend. X: Did not start intervention. Y: Drop out due to disease progression. Z: Class not offered due to COVID-related university closure. Blanks indicate that the program was completed (for participants who attended 12 sessions in under 14 weeks). A total of 100 classes were offered.

**Table S2.** Self-reported PA from weekly activity journals.

| n=7 | **Mean (SD)** | |
| --- | --- | --- |
| **Frequency (weekly)** | 7.26 (2.20) | |
| **Intensity (RPE 1-10)** | 3.63 (0.99) | |
| **Time (minutes)** | 261.68 (79.23) | |
| **Type (weekly)** | **Mean (SD)** | % |
| **Cardiovascular** | 1.18 (1.50) | 16.21 |
| **Resistance** | 1.34 (1.52) | 18.45 |
| **Stretching** | 0.51 (1.25) | 7.05 |
| **ENPAL Class** | 0.94 (0.31) | 13.00 |
| **Daily life PA** | 3.29 (1.62) | 45.29 |

RPE = rating of perceived exertion. Daily life PA is any PA done in as part of daily life tasks (e.g. housework). Percentages represent recorded frequencies for each activity divided by the total number of all activities.

**Table S3.** Pre- and post-intervention physical function measures.

|  | | **Pre** (n=10) | **Pre** (n=3) | **Post** (n=3) |
| --- | --- | --- | --- | --- |
| **BMI (kg/m^2^)** | | 28.5 (5.5) | 26.8 (1.1) | 27.2 (2.1) |
| **Grip Strength (kg)** | **Right** | 32.9 (10.2) | 35.3 (13.0) | 36.8 (12.5) |
|  | **Left** | 31.9 (12.1) | 34.2 (18.8) | 34.7 (16.0) |
| **30s Sit to Stand (repetitions)** | | 12 (6.0) | 15.3 (3.5) | 17.3 (1.5) |
| **Shoulder ROM (degrees)** | **Right** | 152 (14.2) | 151 (5.5) | 152 (1.3) |
|  | **Left** | 151 (16.3) | 156 (5.8) | 150 (8.7) |
| **Sit and Reach (cm)** | | 18.6 (10.8)*^a^* | 15.7 (12.6) | 18.1 (9.6) |
| **1-Leg Balance (seconds)** | **Right** | 20.5 (19.5) | 27.9 (22.1) | 35.2 (17.0) |
|  | **Left** | 22.4 (16.3) | 33.2 (20.5) | 33.6 (19.8) |
| **6MWT Distance (m)** | | 505 (84.0) | 593 (71.2) | 618 (50.0)*^b^* |

All outcomes are presented using mean and standard deviation in brackets. ROM = range of motion. 6MWT = 6-minute walk test. The 6MWT was limited to a single trial to reduce participant burden and the likelihood of eliciting negative symptoms such as shortness of breath. *a: n=9. ­b*: n=2.

**Table S4.** Frequency of information provided to participants during ENPAL nutritional consults.

| n=8 | **Category** | **RD suggestion** | **n** | **%** |
| --- | --- | --- | --- | --- |
| **Recommendations** | Food Choices | Balanced meals | 4 | 50 |
|  |  | Eating every 2-3 hours | 1 | 12.5 |
|  | Energy Intake | Increase | 5 | 62.5 |
|  |  | Maintain | 2 | 25 |
|  | Protein | Increase | 8 | 100 |
|  | Fat | Increase | 4 | 50 |
|  |  | Maintain | 1 | 12.5 |
|  |  | Healthy fats | 1 | 12.5 |
|  | Carbohydrates | Increase | 1 | 12.5 |
|  |  | Maintain | 3 | 37.5 |
|  | Supplements | Nutrient dense nutritional supplement as tolerated | 5 | 62.5 |
|  |  | Unflavored protein powder | 7 | 87.5 |
| **Issues Discussed** | Diarrhea |  | 3 | 37.5 |
|  | Poor appetite |  | 4 | 50 |
|  | Dry mouth |  | 3 | 37.5 |
|  | Constipation |  | 1 | 12.5 |
|  | Mouth sores |  | 1 | 12.5 |
|  | Dry lips |  | 1 | 12.5 |
| **Interventions** | Diarrhea | Imodium | 3 | 37.5 |
|  | Poor appetite | Eat small amounts frequently | 5 | 62.5 |
|  |  | High calorie/protein diet as tolerated | 4 | 50 |
|  |  | Nutrient dense nutritional supplement as tolerated | 4 | 50 |
|  |  | Unflavored protein powder | 5 | 62.5 |
|  | Dry mouth | Frequent use of water | 3 | 37.5 |
|  | Mouth sores | Frequent use of water | 1 | 12.5 |

RD = registered dietitian. Numbers in the rightmost column represent the percentage of nutritional consults where specific information was provided according to the nutrition case report forms.

**Table S5.** Frequency of information provided to participants during ENPAL palliative symptom management consults.

| n=8 | **Category** | **Details** (if applicable) | **n** | **%** |
| --- | --- | --- | --- | --- |
| **Issues Discussed** | Pain | | 6 | 75 |
|  | Nausea/emesis | | 2 | 25 |
|  | Cough | | 3 | 37.5 |
|  | Mood (depression, anxiety, wellbeing, other) | | 5 | 62.5 |
|  | Fatigue | | 5 | 62.5 |
|  | Delirium | | 1 | 12.5 |
|  | Goals of care | | 5 | 62.5 |
|  | Dyspnea | | 6 | 75 |
|  | Insomnia | | 5 | 62.5 |
|  | Appetite | | 3 | 37.5 |
|  | Constipation | | 5 | 62.5 |
|  | Other | Quit smoking (1), diarrhea/rash (1) | 2 | 25 |
| **Interventions** | Opioids | | 1 | 12.5 |
|  | Adjuvant Analgesics | | 5 | 62.5 |
|  | Anti-emetics and Neuroleptics | | 0 | 0 |
|  | Laxatives and Bowel Medications | | 3 | 37.5 |
|  | Goals of Care/Goals of Care Designation | Introduced concept | 3 | 37.5 |
|  |  | Reviewed previous decisions/discussions | 4 | 50 |
|  | Mood-related | Referred to psychosocial oncology | 2 | 25 |
|  | Other | Reviewed medications/other health | 3 | 37.5 |

Numbers in the rightmost column represent the percentage of palliative symptom management consults where specific information was provided according to the palliative symptom management case report forms.

**Table S6.** Participant quotes from semi-structured interviews.

| **Theme 1: A Multimodal Program is Feasible** |
| --- |
| **Key Quotes** |
| *[The assessments] went really, really well. I, that’s what got me definitely hooked onto the program, was the assessment. Um, it was very simple to fill out all the questions and I had no problems whatsoever. I loved the tour, I liked um, being able to walk around the Oval and see where I would be and, I was just in my glory. I was so, so happy. Yeah. Yeah. I was very, very comfortable. Yeah. […] and the questions, the questions were very simple. Very, very simple.* ***P5.*** |
| *The ENPAL program was just absolutely amazing. As soon as I walked in the door, I felt extremely welcome, very relaxed, very comfortable […] I’ve never experienced anything like that before, so, it was a really good support system for me at that time. Especially just getting out of the hospital.* ***P5.*** |
| *Um, the program I think is really, really good because it’s okay to our, really, what I can do it, so, mm, the timing is excellent. It’s not too long and it’s not too short. We all enjoy, and I’m not watching and looking at the clock like oh, I need to go home, no, no, I’m never do that, I enjoy 100% doing the exercise.* ***P2.*** |
| *Oh, they [the instructors] were marvelous. They were really marvelous. And uh, I think their energy and uh, with the way they conducted themselves […] it really became a nice labour to the setting itself, so […] I think they are a great part of having the successful program like that […] it’s the trainers. The way the kind of aesthetic they invoke on the setting and uh, also their expertise, understanding, uh, that definitely, uh, played an important part.* ***P8.*** |
| *To be aware that he’s [the Doctor] there, and we had a good chat and there was all, there was nothing directly relevant because I don’t have any pain […] we established that there is such a thing as the pain clinic, that there is real serious expertise involved, uh that if I get to that situation that I can expect some skill.* ***P4.*** |
| **Additional Quotes** |
| *Exercise Progression: The exercises that I did to start with were right perfect and the exercises that um, [our instructor] copied to me in the email are more challenging and it will take a while to get back into those ones. So, you don’t feel like you’re stagnant, you’re just doing the same thing over and over and over again, you’re working towards something. So, I think that’s really good that they’re doing that, and giving you an incentive to continue at home and improve.* ***P10.*** |
| *Class Frequency: To me, the weekly, coming here once a week was absolutely fine for me. Um, like especially nowadays with me having so many other appointments. And plus I’ve always done my own routine at home, so I just threw in some of your routine stuff as well at home, so. Yeah. But, once a week was great, for me.* ***P1.*** |
| *Class Variety: Getting back to the more variety, uh and different routine, but uh the exercises that you provided were good, like like you know, were very helpful. Just too routine, for me. Same thing over and over.* ***P1.*** |
| *Individualization: So, that’s uh, and that’s good that the person, the trainer understands the different kinds of exercises and the different alternatives to exercises. So, if you cannot do this exercise then the other alternative, it’s so and so. So, that’s that’s a helpful thing.* ***P8.*** |
| *Nutrition: Yeah, and I think it because uh, there’s a lot of uh, um, regarding uh, nutrition when it comes to cancer patients, so I think it’s an important part, an addition to the program to have that part, as part of the program.* ***P8.*** |
| **Theme 2: The Value of a Multimodal Program** |
| **Key Quotes** |
| *So overall, I’m really happy that I’ve been a part of it, it’s been good for me, it’s caused my quality of life to be better […].* ***P4.*** |
| *Well, I think it’s uh, definitely the thing that like it helped to get you back to feeling your normal self again. And physical activity. And that’s ultimately um, cancer is, is in many cases, in my case, it’s not a treatable thing. So, it’s just that, at least for, um, some time, uh, so, the goal here is to enjoy life as it is, as normal as it as possibly can.* ***P8.*** |
| *At the time when I started, I wasn’t sure even I can um, do the exercises, or jump on a treadmill, or go back again and do some weightlifting or the different exercises. Especially I just came out of the hospital and you know it was downhill completely. So, the program actually helped me to get back to the normal and realize that I can still, with the medication that I’m taking, get my energy level up and um, uh, get my muscles to move again and actually, I have I think it somehow made me realize that exercise is a better is a good treatment to the side effects that I have from that medication that is primarily the muscle soreness that I have.* ***P8.*** |
| *So, we all know we have the end stage of cancer and and like we will think about what happened to to to my friend, what happened to them why they are not coming. So, I think it’s a social life, I got some social life back […] I just feel it’s so important to have a group of peoples to work together, and we are on the same, we are on the same boat.* ***P2.*** |
| **Additional Quotes** |
| *Assessment: Probably the most important one was actually the original assessment. Um, I had been largely avoiding doing things cause I would immediately run out of breath. […] The assessment showed me that uh you can run out of breath and keep on going. Okay, and doing things, um, so that was probably the most important thing looking back, was getting started on doing things.* ***P4.*** |
| *Education: Well, it was actually a good, uh, a good part in addition to the actual discussion of the mechanism of the exercise, why we’re doing the exercise. Uh, so, um, some part of the education that we had it regarding ah, it was also regarding a bit of uh, uh, I think motivation as well […] Uh, like it was, it adds up to the whole picture, they were really, really very helpful.* ***P8.*** |
| *Fatigue/Energy: I found after the exercise, it’s funny, after the exercise at the [Thrive], I felt more energetic than I did when I came in.* ***P10.*** |
| *Positive Outlook: Um, I was thinking because you know, I have this situation you know, I have this uh, I have the cancer and all that, you know. And maybe I cannot do it, right? So, yeah, it’s improved me a lot. And even the way I think, it’s helped it’s given me focus to be more positive, you know, you can do it, it will, you know, it will come to pass [laughing].* ***P9.*** |
| *Functional ability in daily life: But, um, as I was starting treatment, we have a walk-out basement. I would not go downstairs more than once a day, max. I think of nothing of running up and down the stairs now.* ***P4.*** |
| *Nutrition: I don’t eat much. And she let me know to um, what I should use, yeah, I I actually, during that time I lose weight when I do exercise and I have, I followed her instructions, I took some Ensure, if I couldn’t eat I take 1 bottle of Ensure a day and it helped me.* ***P2.*** |
| **Theme 3: How to Improve a Multimodal Program** |
| **Key Quotes** |
| *[…] The exercises, it would be nice if you could see them online, um, I know there’s apps that you can get where they have exercises online, but it would be nice to have the one specific for you online […] you know, just in case you forget when you go home and you haven’t done them, you know, you’ve only done them once, then it’d be nice to watch somebody do them.* ***P10.*** |
| *I think what would be missing, um, would be, which is surprising, is having a one-on-one either counsellor or psychiatrist dealing with the issue itself. Um, I never had anybody um, talk about the cancer, basically […] I would like to see them introduce and have a counsellor in there [in ENPAL]. I think talking about the cancer and about their feelings, their emotions.* ***P5.*** |
| *[…] Doing them at home, I wasn’t as motivated. Um, as opposed to being um, at the university, um, I loved the outing and I loved getting out. That’s what I looked forward to. And at home, I’m going oh, I’m staying at home, I’m in the basement, oh, I’m not getting out, I’m not talking to anybody. So, I found that very difficult doing them at home. Cause I love the interaction of getting up, getting dressed, and it gets you motivated, it gets you up and going.* ***P5.*** |
| **Additional Quotes** |
| *Barrier (Appointments): Uh other appointments, like especially like for this week, like, too many appointments. Like I just get appointment out […] And so, your exercise class would be the last on my list right now to attend, so. I got cataract surgery coming up, I got dental surgery coming up, and- And my cancer therapies so.* ***P1.*** |
| *Additional class times: Um, it would be nice, like I know you offer it twice a week, I know people sometimes have certain days they can go and can’t go.* ***P10.*** |

Quotes are arranged according to themes in presented in results. Quotes within results are classified as key quotes, while additional quotes follow to provide further depth.
